# Periodontal disease in a young Romanian convenience sample: radiographic assessment

**DOI:** 10.1186/s12903-019-0774-9

**Published:** 2019-05-29

**Authors:** Simona Ioana Hategan, Angela Ruth Kamer, Cosmin Sinescu, Ronald George Craig, Anca Jivanescu, Andrei Mihai Gavrilovici, Meda-Lavinia Negrutiu

**Affiliations:** 1 Department of Prosthodontics, Faculty of Dental Medicine, University of Medicine and Pharmacy “Victor Babeş” Timisoara, Bd. Revolutiei din 1989, Nr.9, 300041 Timişoara, Romania; 2 Department of Periodontology and Implant Dentistry, New York University, College of Dentistry, 345 East 24th Street, New York, NY 10010 USA; 3 Department of Basic Sciences and Craniofacial Biology, New York University, College of Dentistry, 345 East 24th Street, New York, NY 10010 USA; 4 Department of Prostheses Technology and Dental Materials, Faculty of Dentistry, University of Medicine and Pharmacy “Victor Babes” Timisoara, Bd. Revolutiei 1989, Nr.9, sc.C, et.IV, 300070 Timisoara, Romania

**Keywords:** Prevalence, Periodontal disease, Radiographic assessment, Bone loss

## Abstract

**Background:**

The goal of this study was to determine the distribution of periodontal disease in a population seeking oral rehabilitation in a Romanian prosthodontics department and to identify the factors associated with each type of periodontal condition.

**Methods:**

The study population consisted of patients presenting consecutively to the Prosthodontics Department of the Faculty of Dental Medicine, Victor Babeş University of Medicine and Pharmacy, Timisoara. The diagnosis and classification of periodontal conditions, as well as dental pathologies and conditions, were based on examination of panoramic radiographs. A standardized questionnaire was administered to obtain socio-demographic characteristics (age, gender, ethnicity, education, residency, marital status), medical history, dental/periodontal history (family history of periodontal disease), and behavior (smoking, brushing, flossing and regular cleaning).

**Results:**

Among subjects presenting to the Prosthodontics department, only 34.2% were periodontal disease-free and 65.8% had periodontal disease, of which 11.4% had aggressive periodontitis. In univariate models, age, education, marital status, smoking, and tooth number were associated with chronic periodontitis. Age, education, family history, smoking, and tooth number were associated with aggressive periodontitis. However, in a multivariable model, only age, tooth number and family history were significant.

**Conclusions:**

This study found a high prevalence of periodontal disease in patients seeking oral rehabilitation from the Prosthodontics department. Age, tooth number and family history of periodontal disease were associated with the type of periodontal disease. These results suggest the need for periodontal examination prior to prosthetic oral rehabilitation in this population.

## Background

Prosthodontics and periodontics, while different specialties, are intricately interdependent. The prosthetic restoration and replacement of teeth should be performed and supported by teeth with healthy periodontal tissues to insure a lasting restoration [1–3]. In general, periodontal evaluation and treatment is indicated prior to prosthodontic reconstruction. It would be ideal that most patients referred for prosthodontic reconstruction have already undergone periodontal treatment and therefore, their periodontal condition would be sound. However, one study reported that among patients seeking prosthodontic treatment, 5% had healthy periodontium, 3% gingivitis, and 92% mild to severe periodontitis [4]. This study, however, included only elderly subjects recruited from multiple sites including dental schools, urban and rural private offices. No studies on the incidence and prevalence of periodontal diseases were reported from Prosthodontics departments. Therefore, inference regarding the prevalence of periodontal disease in a population seeking treatment in a Prosthodontics department setting cannot be made. To date, there has been no known study that described the periodontal disease distribution in a Prosthodontics department.

The goal of this study was to determine the distribution of periodontal disease in a population seeking oral rehabilitation in a Romanian Prosthodontics department. Since limited studies [5] and anecdotal data suggest that periodontal disease prevalence in young Romanian people is high compared to USA or Western Europe, we focused our investigation on young people. Smoking, a family history of periodontal disease and the existence of tooth related diseases/conditions, as well as defective restorations, are risks for periodontal disease [6, 7]. An additional goal was to determine factors that are associated with each type of periodontal condition. It is our hypothesis that people seeking treatment in our Prosthodontics department would have low prevalence of periodontal disease and smoking, the number of caries, fillings, roots and crowns would be associated with chronic periodontitis. We further hypothesize that family history of periodontal disease would be associate with aggressive periodontal disease.

## Methods

### Participants

This study population consisted of patients presenting consecutively to the Prosthodontics Department of the Faculty of Dental Medicine, Victor Babeş University of Medicine and Pharmacy, Timisoara. These subjects were enrolled in the period from 2013 to 2016, consented to our study, and fulfilled our research criteria. The study design was approved by the University Ethics Committee. After reviewing the study written protocol, the patients signed their written participation agreement – “The Patient agreement on participation in medical research.” An informed consent form was signed by each participating subject prior to entrance into the study.

In general, patients seeking prosthodontic treatment were referrals from other school departments (approximately 80%) or were self-referred. Annually, approximately 800–900 patients of all ages are seen in the Prosthodontics Department. Subjects were included in the study if they were age ≤ 42, and were not edentulous. Subjects were excluded if they had a history of uncontrolled hypertension, diabetes, radiation, and drug use.

The diagnosis and classification of the periodontal conditions, as well as dental pathologies and conditions, were based on panoramic radiographs. The radiographs were visually evaluated by two calibrated periodontists (AK, RG) and examiners (AK, SH). The radiographs were rated as optimal quality since the information provided was sufficient to obtain diagnostic information.

The classification of periodontal disease used modified published criteria [8]. When there was disagreement regarding diagnosis, a diagnostic consensus was reached after a discussion. The diagnostic criteria were based on the degree of interproximal bone loss. Interproximal bone loss was measured as the distance between cemento-enamel junction (CEJ) and bone crest level (BL). When crowns or fillings were present, the cavosurface margin was used as a reference point. The subject was classified as being periodontically normal (NL) if no visible interproximal bone loss (approximately 10%) was observed. Aggressive periodontitis (AgP) was assigned if there was ≥50% of interproximal bone loss on at least two permanent teeth, including first molars and/or incisors (AgP), and chronic periodontitis (CrP) was defined if the interproximal bone loss did not reach 50% on at least two teeth [9].

Using the radiographs, the number of the existing teeth (excluding the root rests) and root rests (≤half crown) were counted. Teeth with fabricated crowns (no pontics), endodontic treatments (radiopacity of the teeth root canals), fillings (radiopacities on crowns or teeth roots), carious lesions (radiolucencies penetrating the dentin on crowns or teeth roots), and periapical lesions (radiolucencies surrounding the tooth root) were counted. If two or more fillings or carious lesions were present on a tooth (i.e. one mesial and one distal) only one count/tooth was considered.

A standardized questionnaire [10] was administered to obtain information on the socio-demographic characteristics of the patient (age, gender, ethnicity, education, residency, marital status), medical history, dental/periodontal history (family history of periodontal disease), and behavior (smoking, brushing, flossing and regular cleaning). Smoking was defined as Never, Past and Current for patients who currently smoke. Since only eight subjects were ex-smokers, they were added to never smokers forming the non-smoker category.

Most of the panoramic radiographs were taken using Soredex Cranex - 3D Panoramic digital X-ray machine (Soredex, Tuusula, Finland) and Promax 2D digital X-ray device (Planmeca, Helsinki, Finland). All the panoramic radiographs included in the study used standardized protocols concerning the calibration of the radiological equipment, correct positioning of the patient, and image acquisition procedures. Good image quality was assured by using a standardized workflow protocol that was respected for both digital X-ray machines. Correct patient positioning was assisted by a triple laser beam system represented by the three cephalometric lights: Frankfurt-horizontal plane positioning beam, midsagittal light and the focal layer positioning beam. The patient’s head position was placed according to the manufacturer’s specifications. In each case, a bite block was used. To minimize vertical distortions, the occlusal plane was at 6-degree angulation to the horizontal plane. Horizontal plane distortion was minimized by placing the patient’s head in a position in which the sagittal plane of the patient’s head passed through the middle of the bite block. In addition to these panoramic radiographs, we also included 3 panoramic radiographs of optimal quality that were acquired using older X-ray machines.

The general data protection regulations concerning patient’s confidentiality were respected according to the General Data Protection Regulations. Confidentiality and integrity of all the patients’ personal data was maintained by ensuring anonymity through numerical coding of the processed data.

#### Statistical analysis

Statistical analyses were performed using SPSS version 24, Chicago IL. Continuous and categorical data are presented as means and standard deviation and percentages.

The analysis was performed in three steps: First, the distribution of each periodontal condition (NL, CrP, AgP) was determined by chi-squared test **(**χ^2^ test**).** Second, we used one-way analysis of variance (ANOVA), Kruskal-Wallis and [chi] to test whether there were differences in demographic characteristics (age, years of education, gender, residing community, marital status), oral conditions (number of teeth, roots, carious lesions, fillings, endodontic treated teeth, periapical lesions), behavior (smoking) and family history of periodontal disease among the periodontal conditions. The upper vs. lower tertiles were used to dichotomize age and tooth number variables into “low” and “high” groups (cut-points for age and teeth were 31 and 23). These parameters (age, years of education, gender, residing community, marital status, oral conditions) may affect the periodontal condition. Therefore, in the third step we used multinomial, multivariable logistic regression to model the association between various characteristics/parameters and the types of periodontal conditions. In these analyses, the type of periodontal condition (NL, CrP, AgP) was the dependent variable and the parameters defined above were the independent variables. First, we constructed univariate models. The variables significant at *p* = 0.1 were sequentially included in a multivariable model. The final model included all the variables that were significant at *p* ≤ 0.05 in the multivariate model.

The magnitude of association was expressed in odds ratios (ORs) and their 95% confidence interval (CI). In addition, we repeated the above analyses using “No periodontal disease” and “periodontal disease” as the dependent dichotomized variable. In these analyses, the parsimonious model was selected after backward-elimination modeling with a significance level for retention of *p* < 0.2. The total number of significance tests is large; however, being an exploratory analysis, an unadjusted 5% level of significance was used [*p* ≤ 0.05 (two tails)].

## Results

### Characteristics of the population

Table 1 shows the characteristics of the population included in our study. The mean age was 29 (SD = 5.6) with 7 subjects aged ≤20, 30 subjects aged < 20 ≥ 25, 47 subjects < 25 ≥ 30, 39 subjects < 30 ≥ 35, 25 subjects < 35 ≥ 40 and 1 subject 42 years old. Mean years of education were 12.81 with 32% being unskilled, 28% skilled and 40% professional.

**Table 1:** Characteristics of the population

Demographic data	n	Mean (SD) or %	range.
Age [mean (SD)]	149	29.39 (5.6)	18-42
Male/Female (%)	149	51.7/48.3	
Ethnicity: Romanian/Serbian/Hungarian (%)	148	83.2/7.4/8.7	
Married/Unmarried (%)	146	55.5/44.5	
Urban/rural (%)	146	78.1/21.9	
Years of education (mean (SD)	126	12.81 (3.52)	4-22
Family history of periodontitis (%)	123	43.1/56.9	
Systemic health findings (%)			
Yes/No	80	10/90	
Behavior characteristics			
Smoking YES/NO (%)	125	40.8/59.2	
Brushing (%)	78		
never		7.7	
once/week or month		7.7	
once/day		84.6	
Flossing (%)	77		
YES		24.7	
NO		75.3	
Cleaning (%):	77		
Never		22.1	
>1year<2 years		28.6	
>2 years		9.1	
<1 year		40.3	
Radiographic findings [mean (SD)]			
Tooth number	149	24.93 (5.62)	4-32
Root rest number	149	1.34 (2.36)	0-17
Carious lesions	149	3.54 (2.87)	0-13
Fillings	149	3.91 (4.20)	0-16
Prosthetic crowns	149	1.18 (2.15)	0-10
Endodontic treatment	149	2.10 (2.56)	0-12
Periapical lesions	149	1.28 (1.47)	0-7

n = the number of subjects included in the study. Differences in the n denote differences in the number of subjects responding to that specific question. Mean (SD)=mean and standard deviation, %=percentage of subjects. For example, tooth number: n=149 denote that 149 subjects had their teeth counted; the mean and SD for these subjects were 24.93 and 5.62 respectively; the range of tooth number was 4-32. For gender, among the 149 subjects, 51.7% were male and 48.3% were female. Carious lesions, fillings, crowns, endodontic and periapical lesions are the number of teeth count to have these lesions

Ethnically, the majority were Romanian (83.2%) with Hungarian and Serbian constituting less than 10%. Females and males were approximately equally distributed. Most of them lived in an urban environment (78.1%), with more than half of them being married (55.5%). When we compared subjects responding to oral behavior questions (*n* = 77) vs. those who did not, we found that the responders had less teeth and more dental roots (mean 23.79 vs 26.03 and 1.74 vs 0.96; *p* = 0.0.02 and 0.05). There was no difference among the diagnosis categories. Among the 80 subjects providing data on medical history, eight of them reported medical problems: 1 reported allergies, 1 anemia, 1 epistaxis, 1 hypertension and 3 hepatitis B. Subjects included in the final regression model tended to be older [30.03 (SD = 6.03) vs 28.25 (SD = 4.7); *p* = 0.07) and had fewer teeth [(24.16 (SD = 6.10) 26.27 (SD = 4.39); *p* = 0.03]. However, they presented with no difference in education, gender, number of root rests, teeth with fillings, caries, crowns, endodontic or periapical lesions. More subjects with aggressive periodontitis were included (14 vs 3 subjects).

### Prevalence of periodontal disease

Among subjects presenting to the Prosthodontics department, only 34.2% were periodontal disease free. 65.8% had periodontal disease, among which 11.4% had aggressive periodontitis.

### Characteristics of the subjects by periodontal condition

Table 2 shows the characteristics of the subjects in each periodontal condition. Age, education, family history, ethnicity, smoking and history of scaling and root planing differed among the groups. The tooth and root rest numbers were also different. No statistically significant differences were found in gender, residency, and marital status, brushing, flossing or professional tooth cleaning. They did not differ in the number of teeth with carious lesions, fillings or prosthetic crowns, periapical lesions or endodontic treatment.

**Table 2:** Factors associated with or without the different types of periodontal diseases

	NL	CrP	AgP	Significance			
N	51	81	17	p	p	p	p
				NL/CrP/Ag	NL/CrP	NL/AgP	CrP/AgP
Age (*n*=149)							
Younger ≤31	45 (47.9%)	45 (47.9%)	4 (4.3%)				
Older >31	6 (10.9%)	336 (65.5%)	13 (23.6%)	0.01	0.01	0.01	0.02
Gender							
Female	28 (38.8%)	35 (48.6%)	9 (12.5%)				
Male	23 (29.9%)	46 (59.7%)	8 (10.4%)	0.39	0.19	0.88	0.46
Education (*n*=126)							
High (>9)	37 (36.3%)	55 (53.9%)	10 (9.8%)				
Low (≤8)	2 (8.3%)	15 (62.5%)	7 (29.2%)	0.01	0.02	0.01	0.09
Family history (*n*=123)							
No	32 (45.7%)	36 (51.4%)	2 (2.9%)				
Yes	10 (18.9%)	30 (56.6%)	13 (24.5%)	0.01	0.03	0.01	0.01
Residency (*n*=146)							
Urban	40 (35.1%)	64 (56.1%	10 (8.8%)				
Rural	9 (28.1%)	16 (50%)	7 (21.9%)	0.12	0.82	0.06	0.06
Married (*n*=146)							
Yes	21 (25.9%)	49 (60.5%)	11 (13.6%)				
No	28 (43.1%)	31 (47.7%)	6 (9.2%)	0.09	0.04	0.12	0.79
Ethnicity (*n*=148)							
Romanian	40 (32.3%)	72 (58.1%)	12 (9.7%)				
Serbian	9 (81.8%)	1 (9.1%)	1 (9.1%)				
Hungarian	1 (7.7%)	8 (61.5%	4 (30.8%)	0.01	0.01	0.01	0.12
Smoking (*n*=125)							
No	31 (41.9%)	38 (51.4%	5 (6.8%)				
Yes	10 (19.6%)	30 (58.8%)	11 (21.6%)	0.01	0.04	0.01	0.08
Brushing (*n*=78)							
No	1 (8.3%)	7 (58.3%)	4 (33.3%)				
Yes	21 (31.8%)	38 (57.6%)	7 (10.6%)	0.06	0.19	0.02	0.12
Flossing (*n*=77)							
No	17 (29.3%)	32 (55.2%)	9 (15.5%)				
Yes	5 (26.3%)	12 (63.2%)	2 (10.5%)	0.80	0.69	0.76	0.54
Cleanings (*n*=77)							
No	14 (32.6%)	20 (46.5%)	9 (20.9%)				
Yes	8 (23.5%)	24 (70.6%)	2 (5.9%)	0.07	0.16	0.28	0.03
SC/RP (*n*=76)							
No	22 (31.4%)	40 (57.1%)	8 (11.4%)				
Yes	0 (0.0%)	3 (50.0%)	3 (50.0%)	0.02	0 21	0.01	0.06
Tooth number							
High (>22)	48 (45.7%)	53 (50.5%)	4 (3.8%)				
Low (≤22)	3 (6.8%)	28 (63.6%)	13 (29.5%)	0.01	0.01	0.01	0.01
Root rest number	0.75 (1.20)	1.68 (2.94)	1.59 (1.37)	0.03_kw_	0.04	0.01	0.26
Carious lesions	3.12 (2.62)	3.61 (2.75)	4.47 (3.91)	0.38_kw_	0.25	0.28	0.58
Fillings	3.76 (4.38)	4.24 (2.30)	1.47 (2.15)	0.14_kw_	0.26	0.28	0.06
Crowns	0.67 (1.85)	1.44 (2.30)	1.47 (2.15)	0.06_kw_	0.03	0.08	0.84
Endodontic treat	1.61 (2.13)	2.48 (2.82)	1.76 (2.19)	0.15_kw_	0.07	0.88	0.28
Periapical lesions	1.00 (1.67)	1.44 (1.56)	1.41 (1.81)	0.39_kw_	0.16	0.66	0.74

NL=no periodontitis; CrP=chronic periodontitis; AgP=aggressive periodontitis. Continuous data are presented as mean (SD); Categorical data is presented as percentages. P=*p* value; NL/CrP/AgP=comparison among NL, CrP and AgP; NL/CrP=comparison between NL and CrP group; NL/AgP=comparison between NL and AgP group; CrP/AgP=comparison between CrP and AgP group

KW=Kruskal-Wallis. KW test was followed by MW tests

### Periodontal conditions associate with age, tooth number and family history

Table 3 shows that in univariate models, age, education, marital status, smoking, and tooth number were associated with chronic periodontitis. Age, education, family history, smoking, and tooth number were associated with aggressive periodontitis. The final multivariate model shows that only age was associated with chronic, while family history and tooth number were associated with aggressive periodontitis. Tooth number and age tended to be associated with chronic and aggressive periodontitis, respectively. Being older increased the likelihood of having chronic periodontal disease by more than 4-fold [4.55 (Cl95: 1.32–15.70)] and tended to increase the odds of having aggressive periodontitis by 5-fold [5.14 (Cl95: 0.77–34.41)]. Having a family history of periodontal disease increased the likelihood of having aggressive periodontal disease by more than 9-fold [9.42 (Cl95: 1.62–54.85)]. Having few teeth tended to increase the likelihood of having chronic periodontitis by 4-fold [4.15 (Cl95: 0.80–21.58)] while increasing the likelihood of having aggressive periodontitis by almost 13-fold [12.94 (Cl95: 1.54–108.60). In binary logistic regression, only age and tooth numbers were associated with having periodontitis. Being older and having fewer teeth increased the chance of having periodontitis by almost 4-fold (OR = 3.92; 95Cl 1.42–10.84, *p* = 0.01) and 6-fold, respectively (OR = 6.01; 95Cl 1.61–22.34; p = 0.01).

**Table 3:** Odds ratio for significant factors that associate with chronic and aggressive periodontitis in relation to subjects with no periodontitis

	Univariate (*n*=76-149)				Multivariate (*n*=107)			
	Chronic periodontitis		Aggressive periodontitis		Chronic periodontitis		Aggressive periodontitis	
	OR	95%CI	OR	95%CI	OR	95%CI	OR	95%CI
Age								
Young	1	Ref	1	Ref	1	Ref		
Older	6.00	2.30-15.64***	24.37	2.78-33.63***	4.55	1.32-15.70*		
Gender								
Male	1	Ref	1	Ref				
Female	0.62	0.31-1.27	0.92	0.31-2.78				
Education								
High Ed	1	Ref	1	Ref				
Low Ed	5.57	1.22-25.48*	17.15	3.10-95.04***				
Family history								
No FH	1	Ref	1	Ref			1	Ref
FH	2.67	1.13-6.30*	20.80	4.00-108.24***			9.42	1.62-54.85*
Residency								
Urban	1	Ref	1	Ref				
Rural	1.11	0.44-2.75	3.11	0.91-10.39+				
Married								
No	1	Ref	1	Ref				
Yes	2.11	1.02-4.34*	2.441	0.78-7.68				
Ethnicity								
Romanian	1	Ref	1	Ref				
Non Romanian	0.60	0.22-1.68	1.3	0.35-4.76				
Smoking								
No (74)	1	Ref	1	Ref				
Yes (51)	2.45	1.03-5.77*	6.82	1.91-24.41**				
Tooth number								
High (>22)	1	Ref	1	Ref			1	Ref
Low (<22)	6.34	1.80-22.42***	52.00	10.31-262.11***			12.94	1.54-108.60*
Root rest number	1.33	1.03-1.73*	1.32	0.97-1.79+				
Carious lesions	1.07	0.94-1.22	1.17	0.97-1.41+				
Fillings	1.03	0.94-1.12	0.93	0.79-1.08				
Prosthetic crowns	1.23	1.00-1.52*	1.24	0.94-1.63				
Endodontic treatment	1.16	0.99-1.35+	1.03	0.80-1.33				
Periapical lesions	1.24	0.96-1.62+	1.23	0.84-1.81				

+=*P*≤0.10; *=*P*≤0.05; **=*p*≤0.01; ***p≤0.001. Married vs. Single

## Discussion

Our study showed that the prevalence of periodontal disease in young subjects presenting to a prosthodontics department at a university in Western Romania was high. Among the 149 patients seeking prosthodontic rehabilitation, only 34.2% were periodontal disease free while 65.8% had periodontal disease. Among those with periodontal disease, 82.7% had radiographic diagnosed chronic periodontitis and 17.3% had aggressive periodontitis. Our proposed hypothesis of low prevalence of periodontal disease was not supported. Our results suggest that very few people, or none at all, received periodontal treatment prior to seeking prosthodontic rehabilitation. Indeed, in our study, only six of 76 subjects reported having scaling and root planing prior to coming to the Prosthodontics department and 40% professional cleaning.

Our results also showed that age, tooth number, and periodontal family history were associated with the periodontal type. People with chronic periodontitis were older and tended to have fewer teeth compared to those without periodontal disease, while those with aggressive periodontitis had fewer teeth and a family history of periodontal disease. Although there was no information on the reason for tooth loss, periodontal disease is an important cause of tooth loss even in a young population [10–13]. The low number of teeth in the aggressive periodontitis group may be the result of their periodontal condition. The significant association of the family history of periodontal disease with aggressive periodontitis is consistent with other studies across different populations [14–17]. Interestingly, smoking was not significantly associated with either chronic or aggressive periodontitis, as shown in another Romanian study [18]. This is surprising, since one of the most important risks for periodontal disease is smoking [19, 20]. One explanation is the high rate of smoking in Romania across multiple populations including people without periodontal disease [21]. Comparable to Nedo’s study [21], our study showed that 40% of the subjects were smokers. A second explanation is related to the confounding effect of aging. In our analyses, we showed that fewer people smoked in the periodontal-free group. However, in multivariate analysis, the significance of smoking disappeared once age was added to the model, suggesting that smoking varied by age rather than by periodontal groups. And still an additional explanation may be related to recall bias. It is possible that our subjects did not recall or did not report accurately their history of smoking. Sensitivity analysis with ex-smokers included in the smoker category did not change the results. Perhaps this population would constitute a good-targeted population for studying genetic factors that associate with periodontal disease. None of the dental-related parameters were associated with the periodontal conditions. These results are in contrast to most studies showing high correlation between dental conditions and chronic periodontitis [6].

An important question raised by this study is whether this sample represents the younger population of Western Romania? In fact, not only are there no epidemiological studies evaluating the prevalence of periodontal disease in this population, but there are no systematic, epidemiologic population-based studies in Romania. A cross-sectional study of young high school and university students (age 16–35) from urban environments showed that only 0.96% had periodontal disease and 0.48% had aggressive periodontitis [10]. This population certainly does not represent the younger population of Romania. In a study encompassing 143 young and old subjects, the prevalence of chronic and aggressive periodontitis was 60 and 4.2%, respectively [5].

Our study has several limitations, among them, the importance of limited applicability of these results to the younger population of western Romania. This study included only the younger subjects coming to the Prosthodontics department while excluding those with diabetes or other significant medical conditions. The included subjects were less than 42 years of age but the majority of the subjects were older than 30. Other limitations are related to classification and selection bias. The classification of the periodontal and dental conditions was based on panoramic radiographies and no information of the current pocket depth and level of inflammation were available. Although the radiographs are important tools in the clinical diagnosis of the periodontal disease [22–24], panoramic radiography is not reliable to diagnose dental conditions [8]. In addition, misclassification of periodontal disease is possible. For aggressive periodontitis, we used 50% bone loss as threshold criteria [7] while other studies used the bone loss of 30% as the threshold for aggressive periodontitis [20]. Our decision was based on the consideration that compared to other studies, our subjects with aggressive periodontitis were relatively older and presenting a high level of dental conditions. The lack of data from many enrolled subjects certainly introduced a selection bias. The reasons for subjects not responding to all of the questions on the questionnaire are unknown. Lack of information (i.e., family history), or fear of judgment (i.e., smoking, dental behaviors) may be among them. Another limitation was based on a limited sample size. In the final model, the confidence intervals were large, suggesting that the point estimates for ORs may be unstable. Performing a larger study would remediate this issue.

There are several strengths related to this study. This study examined a relatively homogeneous population (young, healthy subjects seeking rehabilitation in a prosthodontics department) allowing us to detect differences even if the number of subjects was limited. The examiners were standardized and calibrated and open discussion solved differences in outcomes.

## Conclusions

In conclusion, this study showed that young people coming to the Prosthodontics department seeking oral rehabilitation have a high prevalence of periodontal disease. Close collaboration between different dental specialties in a university setting is required.

## Abbreviations

2D: bidimensional
3D: threedimensional
AgP: aggressive periodontitis
AK: Angela Kamer
ANOVA: one-way analysis of variance
BL: bone crest level
CEJ: cemento-enamel junction
CI: confidence interval
CrP: chronic periodontitis
KW: Kruskal-Wallis
MW test: Mann-Whitney test
NL: periodontically normal subject
ORs: odds ratios
RC: Ronald Craig
SD: standard deviation
SH: Simona Haţegan
SPSS: software for statistical analysis
